# How Cleft Type and Width Affect the Rate of Secondary Palatal Surgery and Articulation Proficiency in 5-Year-Olds With Cleft Palate

**DOI:** 10.1177/10556656251340816

**Published:** 2025-05-14

**Authors:** Åsa Okhiria, Christina Persson, Monica Blom Johansson, Fatemeh Jabbari, Malin Hakelius, Daniel Nowinski

**Affiliations:** 1 Department of Surgical Sciences, Plastic Surgery, 8097 Uppsala University, Uppsala, Sweden; 2 Institute of Neuroscience and Physiology, Department of Health and Rehabilitation, Speech and Language Pathology Unit, 70712 Sahlgrenska Academy, University of Gothenburg, Gothenburg, Sweden; 3 Department of Otorhinolaryngology, 56749 Sahlgrenska University Hospital, Gothenburg, Sweden; 4 Department of Public Health and Caring Sciences, Speech and Language Pathology, 8097 Uppsala University, Uppsala, Sweden

**Keywords:** cleft width, cleft type, secondary surgery, articulation, PCC, PCC-A

## Abstract

**Objective:**

To investigate the association of cleft type and width with the frequency of secondary palatal surgery, articulation, and velopharyngeal function (VPF).

**Design:**

A cross-sectional study.

**Setting:**

A single multidisciplinary craniofacial team at a university hospital.

**Patients:**

100 patients with a non-syndromic cleft lip and or soft and hard palate born between 2000 and 2015 and treated with a 2-stage palatoplasty. Twenty-one had cleft on the soft and hard palate (SHCP), 17 had bilateral cleft lip and palate (BCLP), and 62 had unilateral cleft lip and palate (UCLP).

**Main outcome measures:**

The impact of cleft type and width on the rate of secondary palatal surgery, the percent of correct consonants (PCC), and PCC adjusted for age (PCC-A), and the composite score for velopharyngeal competence (VPC-Sum) at 5 years of age. Articulation errors were divided into cleft speech characteristics (CSCs) and developmental speech characteristics (DSCs), and the types of errors were compared between the groups.

**Results:**

Neither cleft type nor cleft width was associated with the need for secondary palatal surgery or VPC-Sum. Cleft width but not cleft type was significantly associated with PCC and PCC-A. There were no significant differences between cleft types regarding CSCs or DSCs. The types of errors did not differ between cleft types.

**Conclusions:**

Cleft width predicted PCC and PCC-A and should be included in analyses to identify factors that may impact different outcomes. Cleft type does not seem to be a reliable predictor.

## Introduction

There are several types of cleft deformities. In this study, we define cleft type as one involving the palate. Cleft type is considered one of several factors influencing the rate of secondary palatal surgery^1^ and speech outcomes.^2^ Other factors, such as cleft width,^3–5^ timing of repair,^6–9^ and extent of cleft in isolated cleft palates,^2,10,11^ are also thought to influence speech outcomes. It is common to consider all cleft types when examining surgical outcomes, while, historically, studies often focused on a single selected cleft type when examining speech outcomes. In more recent years, there has been an increase in studies that include all types of cleft palate and describe and compare articulation and velopharyngeal function (VPF) between the cleft types, for example in, Baillie et al., 2020, Butterworth et al., 2023 and Klintö et al., 2022.^2,11,12^

A meta-analysis showed strong evidence that clefts involving only the soft palate were associated with less secondary speech surgery than other cleft types and that bilateral cleft lip and palate (BCLP) were associated with more secondary speech surgery.^1^ It has been found through various studies that wider clefts are more likely to require secondary palatal surgery.^13–15^ Studies controlling for both cleft type and cleft width have demonstrated that cleft width is associated with the need for such surgery rather than cleft type itself.^4,16^

Individuals with BCLP have been shown to have significantly poorer articulation than those with cleft palate only and unilateral cleft lip and palate (UCLP); that is, the more extensive the cleft, the more articulation difficulties.^2,11,12,17–20^ The association between more extensive clefts and velopharyngeal insufficiency (VPI) does not seem to be as linear, and the results are more contradictory.^17,18,20^ It has been shown that individuals with an isolated cleft palate involving both the soft and hard palate have poorer speech outcomes (eg, articulation, VPF, hypernasality, and nasal airflow) than those with clefts in only the soft palate.^2,10,11,21^ The studies demonstrating that individuals with BCLP have poorer articulation than other cleft types did not control for cleft width when assessing articulation ability.

This study aimed to investigate the association between cleft type and width and the rate of secondary surgery and speech outcomes in terms of articulation and VPF. The hypothesis was that wider and more extensive clefts may encounter greater challenges.

The following research questions were posed: (1) How do cleft type and width affect the secondary surgery rate due to healing problems (dehiscence and fistula) and VPI? (2) How do cleft type and cleft width affect articulation and VPF? (3) Are there differences regarding the type of articulation errors between cleft types?

## Methods

The Regional Ethics Committee in Uppsala approved this study (Reference no.: 2017/457).

### Participants

A consecutive series of 356 children born in 2000-2015 with cleft lip and or soft and hard palate and treated at Uppsala University Hospital from birth were reviewed at 5 years of age. The initial inclusion criteria of being non-syndromic, treated at our unit from birth, and treated according to a surgical protocol, including a 2-stage palatal surgery with soft palate closure at 6 months (−2,  + 3 months) and hard palate closure at 24 months (±3 months), were not met by 201 individuals. One hundred and eleven of these did not meet the criteria of 2-stage surgery or the age at surgery. Eighty-six of these individuals had SHCP, of which 74 were treated with 1-stage palatoplasty. Additional reasons for being excluded were syndrome (n = 30), Robin sequence (n = 37), relocating outside our region (n = 15), or being deceased (n = 8). One hundred and fifty-five children met the inclusion criteria, but 55 children were excluded due to (1) missing or incomplete data (n = 40) or (2) having a neurodevelopmental disorder that may affect speech and language development (n = 15). A total of 100 children were finally included: 62 with UCLP (19 girls and 43 boys), 17 with BCLP (6 girls and 11 boys), and 21 with a cleft in the soft and hard palate (SHCP) (15 girls and 6 boys).

Information about the number of visits to a speech and language pathologist (SLP) was available for 83%. Of those, 40 (48%) had received speech intervention. On average, the children had 12 visits, ranging from 1 to 44. Those who did not receive speech intervention had an average of 6 visits, ranging from 1 to 16. In contrast, those who received speech intervention had an average of 18 visits, ranging from 3 to 44.

Outcome data on the percent of correct consonants (PCC) and VPF in individuals with UCLP was also included in a study investigating the impact of surgical techniques and cleft width on the rate of secondary surgery and VPF.^5^

### Surgical Treatment

The standard procedure at Uppsala University Hospital is to perform lip surgery at 3 months, soft palate closure at 6 months, and hard palate closure at 2 years. Until 2007, intra-velar veloplasty reinforced by the palatopharyngeal muscle^22^ was performed. In 2007, radical muscle dissection was introduced; since August 2009, it has been performed according to Sommerlad.^23,24^ Speech-improving surgery was performed with either a re-repair of the soft palate or a pharyngeal flap. Before speech-improving surgery, all individuals underwent a speech assessment and a nasoendoscopy. Surgical details for the included individuals are shown in Table 1.

**Table 1.** Primary and Secondary Surgery Until 5 Years of age.

	Cleft type		
	UCLP (n = 62)	BCLP (n = 17)	SHCP (n = 21)
Soft palate closure (mean age)	6.8	7.3	6.8
Hard palate closure (mean age)	24.3	24.1	24.3
Cleft width (mean ratio, range)	33% (14-45%)	37% (23-45%)	25% (8-39%)
Dehiscence (soft palate)	2 (3%)	1 (6%)	-
Dehiscence (hard palate)	3 (5%)	2 (12%)	3 (14%)
Fistulas (hard/soft palate junction)	2 (3%)	1 (6%)	1 (5%)
Fistulas (alveolar)	1 (2%)	1 (6%)	-
Speech-improving surgery	6 (10%)	3 (18%)	4 (19%)
Secondary palatal surgery (total)	12 (19%)	6 (35%)	6 (29%)

Age at surgery is given in months. The rates of secondary palatal surgeries are provided in the number of individuals and percent.

Abbreviations: mean ratio, ratio A-A1/T-T1; UCLP, unilateral cleft lip and palate; BCLP, bilateral cleft lip and palate; SCHP, cleft in the soft and hard palate.

### Cleft Width Measurement

Dental study casts were obtained at the time of lip-plasty at 3 months of age for those with UCLP or BCLP and at the time of soft palate closure at 6 months for those with SHCP. Using reference points and linear measurements earlier described,^25–27^ an orthodontist with more than 15 years of experience measured the dental study casts. Using a digital caliper, the cleft width was calculated as the ratio A-A1/T-T1 and measured to the nearest 0.01 mm. T-T1 represents the posterior width of the alveolar arch in the tuber area, and the A-A1 represents the width of the cleft at the level of T-T1. Thirty percent of the casts were randomly chosen for repeated measurements to establish intra-rater agreement. Dental casts were available for 90 individuals. Measurements for the 10 individuals whose dental casts were missing were obtained from an earlier study^4^ using the same methodology and measurer as in the present study.

### Speech Material

At the routine control at 5 years of age, a speech sample was audio-recorded with Zoom H4n or a PC with Soundswell software (Saven Hitech, Stockholm, Sweden) and a condenser microphone (Røde NT4, Sydney, Australia or Philips SpeechMike Classic 6264). The speech material used was the Swedish Articulation and Nasality Test (SVANTE).^28^ Using Praat,^29^ 2 sets of speech material were edited from the recordings: (1) Fifty-nine single words, each including one high-pressure target consonant (plosives /p/, /b/, /t/, /d/, /k/, /g/ and voiceless fricatives /f/, and /s/ in initial, medial and final position, and the voiceless fricative /ɕ/ in initial position); (2) A 9-word string (the first 9 monosyllabic words edited to a string of words with no pause between them) including high vowels (/i:/ and /u:/) for assessment of hypernasality. All edited audio files were randomized and assigned codes to enable blinded assessments.

### Speech Assessment

The assessments involved 3 SLPs with at least 5 years of experience in cleft palate speech, 2 independent SLPs (raters 1 and 2) plus the first author (rater 3). A calibration session was conducted before the speech assessments to ensure equivalent assessments. Prior to the calibration sessions, all raters and transcribers did their individual ratings of hypernasality (13 audio recordings) and transcriptions (4 audio recordings). During the following calibration sessions, if there were disagreements among the SLPs regarding the ratings or transcriptions, the audio recordings were listened to, and consensus between raters was obtained after discussion. All 3 SLPs rated hypernasality, while one of the independent SLPs (rater 2) and the first author (rater 3) performed the phonetic transcriptions of target consonants in words.

A semi-narrow phonetic transcription using the International Phonetic Alphabet^30^ and the symbols for nasal escape, velopharyngeal friction, weak articulation, voicing, devoicing, and active nasal fricative from the extended IPA^31^ symbols for disordered speech were performed by raters 2 and 3. The transcriptions made by the independent SLP were used as the results for all participants. PCC was initially developed by Shriberg and Kwiatkowski in 1982 to evaluate PCC in conversational speech,^32^ and modified versions of PCC in single words have been used to assess articulation in children with CLP.^33–35^ All consonant errors were weighted equally, and based on the transcriptions, PCC was calculated (number of correct articulated target consonants/number of elicited target consonants × 100). The target consonant was scored correct if the place and manner of articulation were correct. In addition, the PCC adjusted for age (PCC-A)^36^ was calculated. In PCC-A, age-appropriate s-distortions such as inter-dental, lateral, supra-dental, retroflex, alveolo-palatal, and palatal production of /s/are scored as correct. When calculating PCC and PCC-A, any signs of VPI were disregarded. As described in the Scandcleft project,^37,38^ articulation errors were divided into cleft speech characteristics (CSCs) and developmental speech characteristics (DSCs). CSCs were subdivided into non-oral errors (glottal or pharyngeal plosive or fricative, nasal for unvoiced stop or fricative, and active nasal fricative) and oral errors (retracted/backed to palatal/velar/uvular place of articulation and double articulation). Errors related to development noted in the present study were velar fronting (a velar consonant is replaced with a dental or alveolo-dental consonant), stopping (a fricative is replaced with a plosive), and voicing errors (difficulties differentiating between voiced and voiceless consonants) and were considered DSCs. Three or more occurrences signified an error. An articulation error could be counted as both a CSC and a DSC.

To assess velopharyngeal competence (VPC), VPC-Sum was used.^39,40^ VPC-Sum is a composite score including (a) perceptual ratings of hypernasality rated on a 4-point scale (0 = normal, 1 = mild, 2 = moderate, and 3 = severe), (b) perceptual signs of VPI from transcriptions (nasal emission and weak pressure consonants), and (c) active non-oral speech errors from transcriptions, used to assess VPC at the word level. The 3 variables generated a score of 0-2 and were added to calculate VPC-Sum 0-6. In the statistical analyses, the results from VPC-Sum will be used according to the interpretation of VPC-Sum: 0-1 = 0: competent, 2-3 = 1: marginally incompetent, and 4-6 = 2: incompetent.

The audio files were listened to through high-quality headphones and could be replayed as many times as needed. Thirty percent of the audio files were randomly selected for re-assessment to calculate intra-rater agreement.

### Statistical Analysis

To determine intra-rater reliability for cleft width measurement, the single measures intra-class correlation coefficient with a 2-way mixed-effects model (ICC) was used. The levels of observed agreement were interpreted according to Cicchetti^41^ as follows:  < .40 is poor, .40-.59 is fair, .60-.74 is good, and .75-1.00 is excellent. Descriptive statistics were used to investigate the inter- and intra-rater agreement for transcriptions and hypernasality, and agreement was calculated as described in the next section. Logistic regression analysis was used to examine the impact of cleft type and cleft width on the rate of secondary palatal surgery. The effect of cleft type and cleft width on PCC and PCC-A was analyzed using linear regression analysis. An ordinal regression analysis investigated the association between cleft type and cleft width with VPC-Sum. VPC-Sum was categorized as 0 = competent, 1 = marginally incompetent, and 2 = incompetent in the statistical models. Possible differences between the cleft types regarding CSCs and DSCs were calculated with the Chi-2 test. A Kruskal–Wallis analysis was used to detect any differences in cleft width between those who had and those who did not have CSCs or DSCs. Statistical analyses were performed in IBM SPSS Statistics, version 29, or R. The level of significance was set at α < 0.05 (2-tailed).

### Reliability

Intra-rater reliability for cleft width measurement was good (.718, 95% CI .475-.860). Inter-rater agreement for hypernasality was calculated as the frequency of (1) agreement between all 3 raters, (2) agreement between 2 raters, and (3) no agreement. The inter- and intra-rater agreement for hypernasality was measured as agreement point-by-point, as was inter- and intra-rater agreement for the transcriptions. Comparisons were made for correctly articulated target sounds, manner of articulation, place of articulation, VPI symptoms, and non-oral articulation. Minor differences between the transcribers that were considered as an agreement were /ʔ and b͡ʔ/ (glottal vs. glottal reinforcement), /ʔ and ∅*/ (*omission of a consonant), /c and k/, and /ɟ and ɡ/. Inter- and intra-rater agreement for hypernasality and the transcriptions are shown in Tables 2 and 3.

**Table 2.** Inter- and Intra-Rater Agreement for Hypernasality.

Inter-rater agreement	At least 2 out of 3 raters agree (%)	92%
	All raters agree (%)	45%
	No agreement (%)	8%
Intra-rater agreement	Rater 1	77%
	Rater 2	85%
	Rater 3	69%

**Table 3.** Inter- and Intra-Transcriber Agreements.

	Inter-transcriber agreement	Intra-transcriber agreement	
Agreement on		Rater 2	Rater 3
Correct/incorrect articulation	91% (58-100)	96% (81-100)	97% (84-100)
Manner of articulation	98% (76-100)	98% (88-100)	100% (96-100)
Place of articulation	89% (65-100)	95% (77-100)	97% (83-100
Symptoms of VPI	93% (70-100)	97% (79-100)	96% (83-100)
Presence of non-oral articulation	100% (70-100)	100% (85-100)	100% (89-100)

Median Percentages (min-max).

Abbreviation: VPI, velopharyngeal incompetence.

## Results

### Secondary Palatal Surgery

Logistic regression analysis showed that cleft type was not associated with the rate of secondary palatal surgery due to dehiscence or fistula or the rate of speech-improving surgery, nor was cleft width; see Table 4.

**Table 4.** Logistic Regression Analyses on How Cleft Type and Cleft Width Were Associated With the Rate of Secondary Palatal Surgery.

	Secondary palatal surgery due to fistula or dehiscence			Speech-improving surgery		
Variable	OR	95% CI	*P*	OR	95% CI	*P*
UCLP (n = 62)	REF		.490	REF		.164
BCLP (n = 17)	1.507	.336-6.760	.593	1.515	.322-7.131	.599
SHCP (n = 21)	2.435	.535-11.079	.250	4.858	.950-24.850	.058
Cleft width	1.033	.952-1.122	.437	1.099	.998-1.209	.054

Abbreviations: UCLP, unilateral cleft lip and palate; BCLP, bilateral cleft lip and palate; SCHP, ceft in the soft and hard palate; OR, odds ratio; CI, confidence interval.

### Articulation

**Figure 1.**
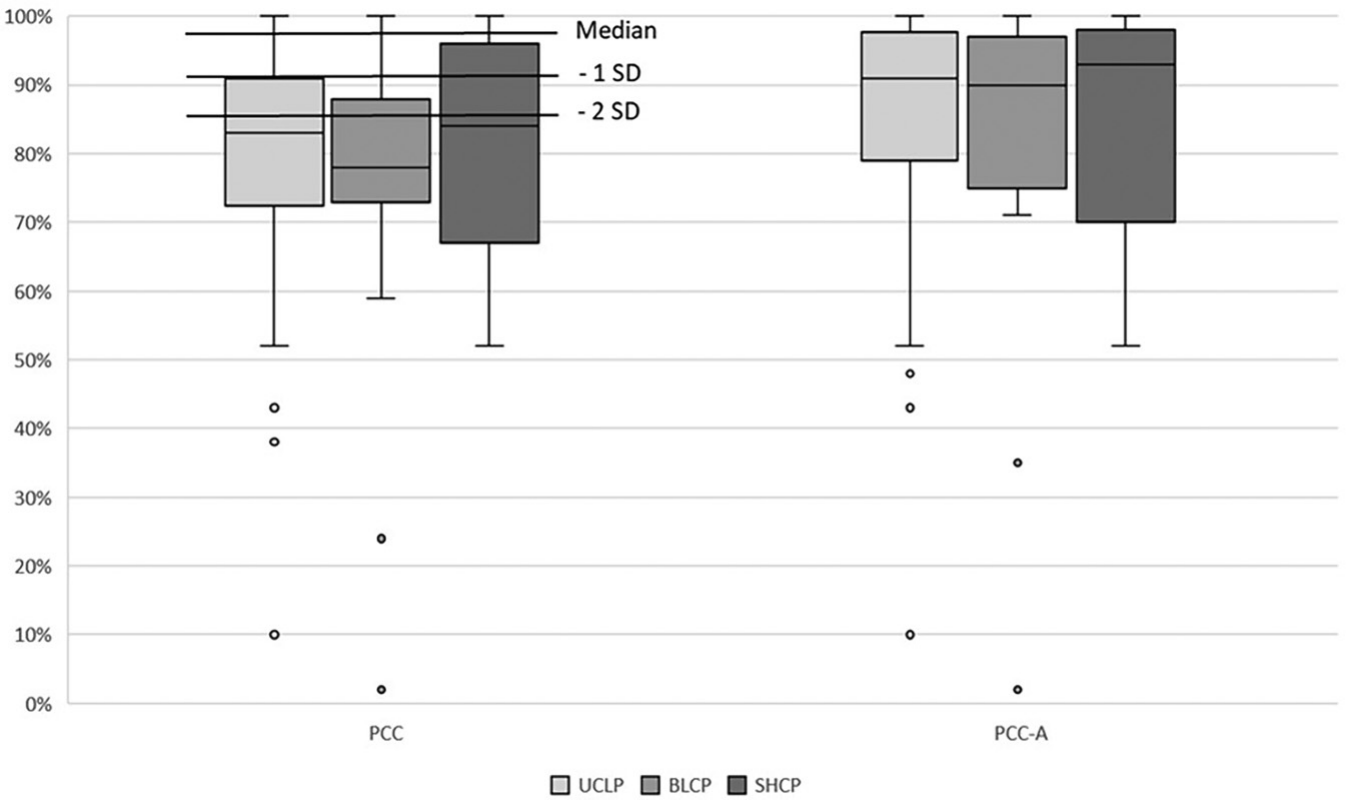
Comparison of median percent of correct consonant (PCC) and PCC adjusted for age (PCC-A) between cleft types. The normative data for PCC is displayed; SD = standard deviation.

Linear regression analysis showed that cleft type was not significantly associated with PCC or PCC-A, but cleft width was, as seen in Table 5. Median PCC and PCC-A are shown in Figure 1.

**Table 5.** Linear Regression Analysis on How Cleft Type and Cleft Width Were Associated With the Percent of Consonants Correct (PCC) and PCC Adjusted for Age (PCC-A).

	PCC			PCC-A		
Variable	OR	95% CI	*P*	OR	95% CI	*P*
UCLP (n = 62)	REF			REF		
BCLP (n = 17)	−3.488	−33.340-6.363	.483	–3.031	–12.945-6.882	.545
SHCP (n = 21)	–3.389	–13.769-5.993	.436	–4.568	–14.511-5.374	.364
Cleft width	–0.676	–1.172 to −0.179	**<**.**01**	–0.604	–1.104 to −0.105	**<**.**05**

Abbreviations: UCLP, unilateral cleft lip and palate; BCLP, bilateral cleft lip and palate; SCHP, cleft in the soft and hard palate; OR, odds ratio; CI, confidence interval, significant p-values in bold.

There was no significant difference for CSCs between cleft types (χ^2^(2) *P* = .869). One or more types of CSCs were present in 59% of all participants (BCLP 65%, UCLP 58%, SHCP 57%). Having more than 1 type of CSC was most common in the BCLP group, and 24% had more than 1 type of CSC compared to none in the SHCP group and 6% in the UCLP group. The frequency of oral and non-oral articulation errors was more common in individuals with BCLP (59% and 24%, respectively) compared to those with UCLP (52% and 15%, respectively) and SHCP (48% and 14%, respectively). Those exhibiting CSCs had a median cleft width of 34% compared to 30% in those not exhibiting CSCs, and the difference was statistically significant (H [1] = 4.2, *P* = .040).

No significant differences between cleft types were found for DSCs (χ2(2) *P* = .531). One or more types of DSC were present in 54% of all participants (BCLP 65%, SHCP 57%, UCLP 50%). It was most common in the SHCP group to have more than 1 type of DSC. 19% in the SHCP group had more than 1 type of DSC compared to only 6% in the BCLP group and 8% in the UCLP group. When analyzing subtypes of DSCs, velar fronting was rare but most common in individuals with SHCP (10%) compared to those with BCLP and UCLP (6% and 3%, respectively). Stopping was marginally more common in individuals with SHCP (14%) compared to those with BCLP and UCLP (12% and 11%, respectively), as was voicing errors (14%) compared to those with BCLP and UCLP (12% and 10% respectively). There was no significant difference in cleft width between those who had (median 34.5%) and those who did not have DSCs (median 31%) (H [1] = 3.731, *P* = .054).

Having both CSCs and DSCs was most common in the SHCP and BCLP groups (48% and 47%, respectively) compared to the UCLP group (27%). The most affected target sound was /s/ in all groups, present in 65% of the BCLP group, 60% in the UCLP group, and 52% in the SHCP group. The s-errors mainly consisted of palatalization of /s/ or inter-dental realization of /s/.

### Velopharyngeal Function

Ordinal regression analysis showed that neither cleft type (UCLP-BCLP: OR 2.651, 95% CI .941-7.470, *P* = .065; UCLP-SHCP: OR 2.402, 95% CI .790-7.301, *P* = .122) nor cleft width (OR 1.332, 95% CI .755-2.351, *P* = .322) was significantly associated with VPC-Sum.

## Discussion

This study investigated the impact of cleft type and width on the rate of secondary palatal surgery, articulation, and VPF. It also sought to determine whether there were any differences in the types of articulation errors between cleft types.

No significant association was found between cleft type or width and the rate of secondary palatal surgery due to dehiscence or fistula or the rate of speech-improving surgery at 5 years of age. In this study, the rate of speech-improving surgery was found to be 19% in the SHCP group, 18% in the BCLP group, and 10% in the UCLP group. These findings seem to contradict the results of some earlier studies that show a higher incidence of speech-improving surgery with more extensive clefts^19,42^ but are consistent with another study that reported speech-improving surgery rates of 18% for SHCP and BCLP groups and 9% for a UCLP group.^18^ The same was shown in Marrinan et al. (Marrinan et al., 1998), who speculated whether the attachment to vomer is crucial. They found a significant difference between palatal clefts with an attached vomer (cleft in the soft palate and UCLP) and palatal clefts without an attached vomer (SHCP and BCLP). The SHCP group in the whole cohort born between 2000 and 2015 constituted approximately another 130 individuals, meeting the inclusion criteria of being treated at our unit since birth. Many of them were excluded because they had undergone 1-stage palatoplasty. The individuals included in the study likely had more severe and wider clefts and were possibly not considered suitable for 1-stage surgery, according to the practice at our unit. This could constitute a selection bias towards more severe SHCP, obscuring a true difference between cleft types. Another possible explanation for the differences in rates of speech-improving surgeries between studies is that different CLP teams most likely have different thresholds for performing or may perform them at various ages. Cleft width has been shown to be associated with the need for secondary palatal surgery.^4,5,16^ Though insignificant, this study also found that a wider cleft tended to be more likely to require speech-improving surgery.

The cleft type was not associated with PCC or PCC-A, and all 3 groups performed substantially lower compared to typically developed children without cleft palate. The median PCC of just above or below 80% was more than 2 standard deviations (SD) below the 97% norm.^28^ A PCC of 91% equals 1SD, which can be considered as age-appropriate. This was reached by 32% in the UCLP group, 29% in the SHCP group, and 18% in the BCLP group. In comparison, another study reported that 65% of the SHCP group, 33% of the UCLP group, and none of the BCLP group reached this level and that the difference between BCLP and SHCP and UCLP was statistically significant.^19^ When calculating PCC-A, the median improved to 91% in the UCLP group, 90% in the BCLP group, and 93% in the SHCP group, indicating that s-errors that are scored as correct in PCC-A account for the greater part of the articulation difficulties. S-errors were present in 65% of the total cohort. Although s-errors are age-appropriate at 5, a more significant proportion of children with CLP experience them compared to the norm population, where s-errors are seen in ∼15% of the children.^28^

No significant differences were observed between the groups when dividing articulation errors into CSCs and DSCs. The most common type of CSC in all groups was retracted articulation to palatal or velar/uvular place, which also was the case in Baillie et al.^11^ and Butterworth et al.^2^ The Scandcleft trials showed that retracted articulation was more common in children who underwent hard palate closure at 3 years than those with hard palate closure at 1 year. Both groups had soft palate closure at 3 to 4 months.^37^ Our unit uses a 2-stage palatal closure with delayed hard palate closure until 2 years of age. This may explain, at least partly, the higher frequency of retracted articulation in our study compared to the studies mentioned above.^2,11^

The number of individuals exhibiting different types of CSCs and/or DSCs varied among different cleft types; however, the types of articulation errors were consistent across these groups. Notably, children with BCLP seem to be more susceptible to CSCs, while children with SHCP appear to be more prone to DSCs.

Overall, the frequency of individuals with articulation difficulties was slightly higher in the BCLP group, which aligns with previous studies.^2,11,12,17–20^ However, they did not differ significantly from the 2 other groups in the present study. Cleft width, on the other hand, was significantly associated with PCC and PCC-A. Cleft width is related to cleft type,^43^ meaning wider clefts are more common in more extensive clefts. However, this does not necessarily translate into clefts involving more structures having less favorable outcomes than those involving fewer structures. Indeed, our results indicate that a narrow but extensive cleft may have more favorable outcomes than a wide but less extensive cleft. Closing a wider cleft can be more challenging, and even if the surgery is successful, it may result in a slightly shorter palate than that of a narrower cleft. This difference can potentially impact the development of articulation. Therefore, we might need to monitor articulation development more closely in cases of wider clefts. Consequently, we must consider the cleft width to make more accurate predictions at an individual level.

One limitation of this study is the small sample size. Larger groups may have yielded other results, and thus, the results should be interpreted cautiously. Another limitation of this study is the lack of hearing status. The impact of hearing impairment on speech outcomes varies among studies, but its impact cannot be ruled out. A significant correlation between the history of articulation and hearing difficulties has been shown,^11,44^ and hearing levels at 18 and 36 months correlated to PCC at 3 years of age.^45^ Contrary to these findings, research has shown that hearing was not linked to speech outcomes at 3^36^ or 5 years old.^20^ Our catchment area is extensive, and many children receive their intervention at clinics closer to their homes. This means that the data regarding the visits to an SLP did not specify the interventions received by the children. The type of intervention may have influenced the results but could not be controlled for.

## Conclusions

This study found that cleft width predicts PCC and PCC-A. However, the relationship between cleft width and speech outcomes has rarely been explored, and further investigation is needed before drawing definitive conclusions. While cleft width did not significantly influence the rate of secondary surgeries or VPF, it should still be considered when examining factors that may affect various outcome measures. The cleft type was not significantly associated with any outcome measurements and may not be regarded as a reliable predictor.
